# Synergistic Effects of Processing Additives and Thermal Annealing on Nanomorphology and Hole Mobility of Poly(3-hexylthiophene) Thin Films

**DOI:** 10.3390/polym11010112

**Published:** 2019-01-10

**Authors:** Min Soo Park, Felix Sunjoo Kim

**Affiliations:** School of Chemical Engineering and Materials Science, Chung-Ang University, Seoul 06974, Korea; vvifro06@naver.com

**Keywords:** polymer semiconductor, processing additive, morphology control, organic field-effect transistor, charge-transport property

## Abstract

Control of the nanoscale molecular ordering and charge-carrier mobility of poly(3-hexylthiophene-2,5-diyl) (P3HT) was achieved by the combined use of processing additives and thermal annealing. Evaluation of four processing additives (1,8-octanedithiol (ODT), diphenyl ether (DPE), 1-chloronaphthalene (CN), and 1,8-diiodooctane (DIO), which are commonly used for the fabrication of organic solar cells, revealed that the nanoscale molecular ordering and, therefore, the charge-carrier mobility, are largely affected by the additives, as demonstrated by spectral absorption, X-ray diffraction, and atomic force microscopy. Thermal annealing selectively influenced the morphological changes, depending on the solubility of P3HT in the additive at high temperature. In the case of CN, in which P3HT can be dissolved at moderate temperature, significant molecular ordering was observed even without thermal annealing. For DIO, in which P3HT is only soluble at elevated temperature, the mobility reached 1.14 × 10^−1^ cm^2^ V^−1^ s^−1^ only after annealing. ODT and DPE were not effective as processing additives in a single-component P3HT. This study provides insight for designing the processing conditions to control the morphology and charge-transport properties of polymers.

## 1. Introduction

Organic and polymer semiconductors have received a lot of attention in the fields of physical and materials chemistry, as well as physics and engineering, due to their interesting charge-carrier dynamics, physical behavior, processing flexibility, and novel applications [1,2,3,4]. With the unprecedented interest in environmental and energy issues, studies of organic solar cells have continued to garner interest for more than two decades [5,6]. Various strategies based on physical chemistry and device physics have been employed to develop high-performance solar cells and transistors, including the development of novel materials and various techniques for device fabrication [2,7]. Notable examples of device engineering include variations in the film deposition [8] and annealing conditions [9,10,11], the use of processing additives and solvent mixtures [12,13,14,15,16,17], as well as pre-aggregation and the formation of nanostructures [18] as effective techniques for enhancing the power conversion efficiency. Among these strategies, the use of processing additives has become an essential step for the development of organic solar cells, as it permits phase separation to form nanoscale morphologies that facilitate ultrafast exciton dissociation and reduce charge recombination [12,13,14,15,16,17].

One of the major challenges in the development of organic electronics is increasing the charge-carrier mobility [1,2,3,4]. Since charge transport in the constituent materials of organic electronics is an essential property, this phenomenon has been widely studied by using organic field-effect transistors as a platform of investigation [19,20]. Charge transport in organic semiconductor films is known to depend strongly on the crystalline structure, molecular orientation, and morphology of the films [21,22]. Most of the commonly used additives for organic solar cells have a boiling point higher than that of the host solvent. The additives are also known to selectively dissolve the electron-accepting component, [6,6]-phenyl-C61-butyric acid methyl ester (PCBM), of polymer:fullerene bulk-heterojunction solar cells. The enhancement in the performance of organic solar cells induced by additives is attributed to the phase separation and changes in the nanomorphology of the blends and the crystallinity of the one component, where the additive acts as a selective solvent for the other component (i.e., PCBM) [14,23,24]. For example, the addition of alkylthiols to the blend solution resulted in significant changes in the absorption and morphology of a P3HT:PCBM film, and the performance of the corresponding solar cell, although the effects on the pure P3HT film were small. In this case, phase separation was only induced when PCBM and thiols were both present in the polymer blend [25]. In another study, thermal annealing was effective for P3HT:PCBM films processed without an alkylthiol, but ineffective with the thiol [13]. However, in a single-component polymer in the absence of PCBM, it remained unclear whether charge transport in the polymer was enhanced or not. In order to better understand the role of the processing additives, the effects of the added component on the morphology and charge transport of the polymer need to be deconvoluted from their effects in blend films. Notably, there has been little focus on the *combined* effects of processing additives and post-treatment on the charge-carrier mobility in single-component polymer semiconductors [26,27,28].

In this study, we investigated the combined effects of various processing additives and post-deposition thermal annealing on the morphology, molecular ordering, and field-effect charge-transport properties of thin films of P3HT. We chose P3HT because of its unique position in terms of long history of research as a benchmarking material in polymer electronics, and availability with quality control for easy comparison and reproducibility. We selected four common processing additives, namely, 1,8-octanedithiol (ODT) [12], diphenylether (DPE) [29], 1-chloronaphthalene (CN) [30], and 1,8-diiodooctane (DIO) [14]. The present objective is to deconvolute the additive effects of phase separation on charge transport in a thin polymer film from blend systems. The annealing temperature in this work is 120 °C, which is compatible with various polymer substrates and processing technology. It is demonstrated that the degree of molecular ordering varies depending on the additives. The temperature-dependent solubility of P3HT in the additives governs the mode of molecular ordering and, therefore, the charge-carrier mobility.

## 2. Materials and Methods

Preparation of Polymer Thin-Films and Organic Field-Effect Transistors: Regioregular poly(3-hexylthiophene-2,5-diyl) (P3HT) was obtained from Rieke Metals, Inc. (Lincoln, NB, USA), and was used without further purification. The vendor-specified molecular weight and regioregularity of P3HT were 50–70 kg mol^−1^ and 91%–94%, respectively. Chloroform, octadecyltrichlorosilane (ODTS), 1,8-octanedithiol (ODT), diphenyl ether (DPE), 1-chloronaphthalene (CN), and 1,8-diiodooctane (DIO) were purchased from Sigma-Aldrich (St. Louis, MO, USA). A highly doped Si wafer with a thermally grown 200 nm-thick silicon dioxide (SiO_2_) layer was used as a gate electrode and a dielectric layer. The substrates were cleaned by sequential ultrasonication in a bath of deionized water, acetone, and isopropyl alcohol (15 min each), and then dried under flowing N_2_. The ODTS-modified SiO_2_ surface was obtained by immersing the clean wafer in a toluene solution of 5 mM ODTS at room temperature for 60 min, followed by annealing at 100 °C for 10 min in air. A 10 mg mL^−1^ P3HT solution was prepared in chloroform. As a processing additive, 2.5 vol % of either ODT, DPE, CN, or DIO was added to the P3HT solution. P3HT thin films were deposited by spin-coating at 2000 rpm for 60 s. The thickness of the P3HT layer was about 60–70 nm, based on atomic force microscopy (AFM) measurement. If necessary, the thin films were annealed at 120 °C for 30 min under nitrogen environment. To complete the field-effect transistors, gold source and drain electrodes (~50 nm) were deposited by thermal evaporation through a shadow mask.

Characterization: UV–Vis spectra were obtained in the range of 300–900 nm by using a V-670 spectrophotometer (JASCO, Inc., Easton, MD, USA). The surface morphology of the thin films was observed by using an atomic force microscope (XE-100, Park Systems, Suwon, Korea). X-ray diffraction (XRD) measurements were performed on a Bruker D8-Advance instrument with a Cu-Kα source. The d-spacing and the mean coherent length (*L*_C_) of the crystalline domains in the P3HT films were estimated from the Scherrer equation: *L*_C_ = *Kλ*/(*B* cos*θ*), where *K* is the dimensionless shape factor (0.9 was used here), *λ* is the incident X-ray wavelength (0.15406 nm), *θ* is the Bragg angle, and *B* is the full-width at half-maximum (FWHM) of the peak after background subtraction and pseudo-Voigt curve fitting. The electrical performance of the devices was characterized by using a HP4156A parameter analyzer at room temperature. The field-effect mobility (*μ*) and the threshold voltage (*V*_T_) of the device were calculated from the saturation regime curves (*V*_D_ = −100 V) by using the equation: *I*_D_ = (*μC*_i_*W*/2*L*)(*V*_G_ − *V*_T_)^2^, where *I*_D_ is the drain current, *W* is the channel width (1 mm), *L* is the channel length (100 μm), *C*_i_ is the capacitance of the gate dielectric (17.3 nF cm^−2^ in this work), and *V*_G_ is the gate voltage.

## 3. Results and Discussion

**Figure 1:**
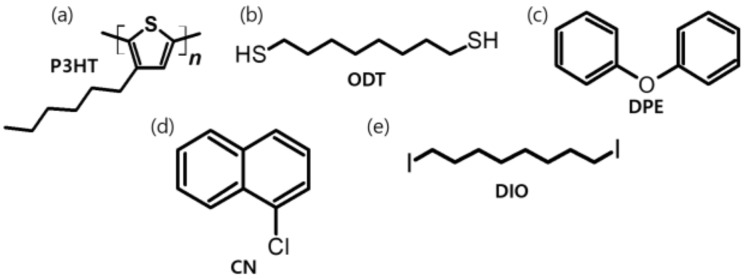
Chemical structures of polymer and processing additives used in this work: (**a**) poly(3-hexylthiophene-2,5-diyl) (P3HT), (**b**) 1,8-octanedithiol (ODT), (**c**) diphenyl ether (DPE), (**d**) 1-chloronaphthalene, and (**e**) 1,8-diiodooctane (DIO).

The structures of the chemicals used in this work are shown in Figure 1. Regioregular P3HT was selected as a model system because it is commercially available, exhibits reasonably good charge-transport properties, and has been intensively studied as a benchmark polymer semiconductor for the development of novel materials and processing methods [31,32,33,34]. From a literature survey, it was found that high boiling point (or low vapor pressure) chemicals have generally been used as processing additives in the fabrication of organic solar cells [17,24]. Among these chemicals, the most widely used ones, i.e., ODT, DIO, CN, and DPE, were selected. Despite the wide use of such additives for materials and device engineering in organic solar cells, there are only a few studies investigating their effects on charge transport in a single-component system [26,35,36]. These commonly used additives are liquid at room temperature and have high boiling points ranging from 259 to 332 °C. They are not volatile at room temperature, as the vapor pressure of the chemicals are lower than 0.03 mmHg at 25 °C. Due to the high boiling point, the application of processing additives in conjunction with thermal annealing should affect the nanoscale morphology and charge-transport properties of the polymer thin films.

**Figure 2:**
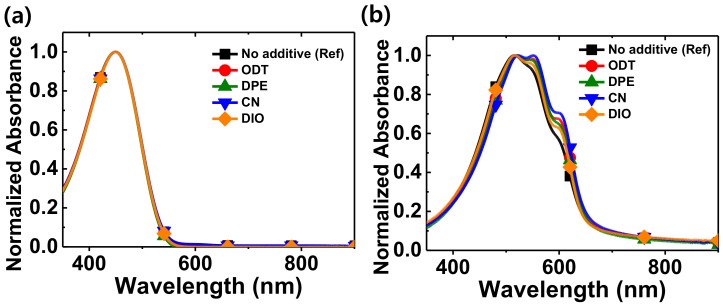
Normalized UV–Vis absorption spectra of P3HT at room temperature: (**a**) absorption in chloroform with and without additives. (**b**) Absorption of thin films processed with and without additives. The films were thermally annealed at 120 °C for 30 min.

The UV–Vis absorption spectra of the P3HT solutions, with and without additives, and those of the thin films spin-coated from the solutions, are presented in Figure 2. The solution spectra showed only one peak at 450 nm associated with the intrachain π–π* transition of P3HT [37]. Despite the addition of 2.5 vol % of the additive, there was no evidence of additional features related to intermolecular interactions, suggesting that the P3HT chain remained molecularly well dissolved without any noticeable charge-transfer phenomenon. The spectra of the P3HT thin films showed a dominant peak at 520 nm (Figure 2b). Unlike the solution absorption spectra, the evolution of two shoulders at longer wavelengths of 550 and 603 nm was observed, indicative of interchain interactions of the P3HT molecules [38]. The relative intensity of the new features increased in the order of DIO, DPE, ODT, and CN.

**Figure 3:**
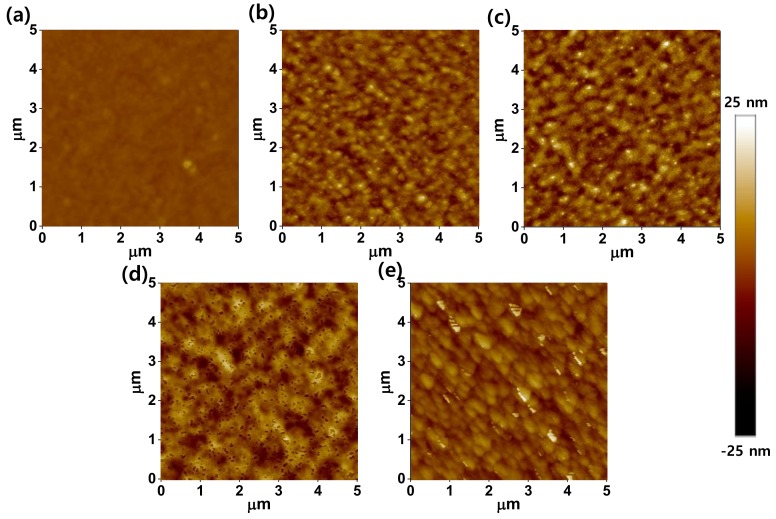
AFM topographic images: (**a**) P3HT films processed without additive, and with (**b**) ODT, (**c**) DPE, (**d**) CN, and (**e**) DIO. The films were thermally annealed at 120 °C for 30 min.

Evidence of molecular aggregation in the thin films due to the processing additives was found from observation of the surface morphology and thin film crystallinity (Figure 3 and Figure S1). The root mean square roughness (*R*_q_) of the P3HT thin films increased from 0.8 nm without any additive to 3.8 nm (ODT), 5.0 nm (DPE), 5.3 nm (CN), and 3.9 nm (DIO), as shown Figure 3. Even though the film becomes rougher (*R*_q_ ~ 3–5 nm) after processing with additives, the height difference is much smaller than the film thickness (60–70 nm), and the P3HT films are still continuous. Since the charge transporting channel is formed on the side of dielectric interface, the charge-carrier mobility would be affected by the aggregation behavior, not by the surface roughness itself.

Similar observations have been reported for other polymer and solvent mixtures [26,35]. The X-ray diffraction (XRD) profiles show the characteristic peak of the (100) plane of lamellar-stacked P3HT at 5.3°–5.4°, which corresponds to a d-spacing of ~1.6–1.7 nm (Figure S1). It is clear that the use of additives enhances the intensity of this peak for the P3HT thin films compared to that for the pristine samples prepared without any additive, suggesting an increase in the degree of molecular ordering in the former [32]. Among the additives, CN and DIO had a more significant impact on the molecular packing in the [100] direction than ODT and DPE. The d-spacing decreased from 1.67 nm for the films without additive to 1.64 nm with ODT or DPE, and to 1.63 nm with CN or DIO. At the same time, the mean coherent length (*L*_C_) of the crystalline domains, calculated using the Scherrer equation, increased from 13.2 nm for the films without additive to 16.7 nm with ODT, 16.8 nm with DPE, 18.2 nm with CN, and 24.8 nm with DIO. Although the peak intensity, d_(100)_-spacing, and the *L*_C_ from XRD may not be absolute measures for determining the charge-transport capability of polymer semiconductors, they are expected to be strongly related to the performance of the thin film devices.

**Figure 4:**
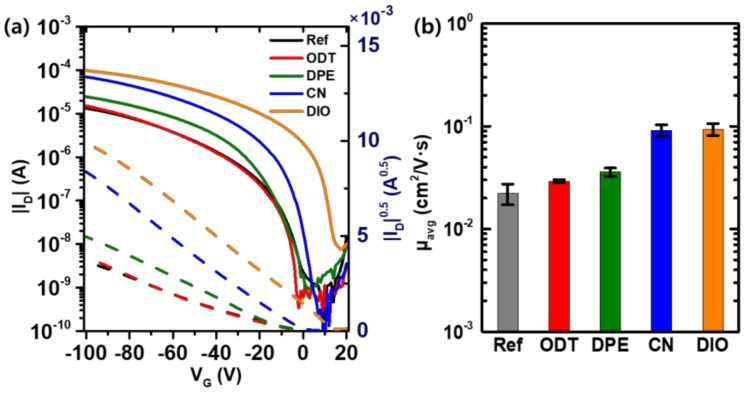
(**a**) Characteristics of organic field-effect transistors (OFETs) based on P3HT processed with various additives. (**b**) Comparison of hole mobility. The films were thermally annealed at 120 °C for 30 min.

In order to investigate the effects of various processing additives on the charge-transport properties of polymer semiconductors, organic field-effect transistors (OFETs) based on the benchmark P3HT thin films were fabricated and evaluated. The representative electrical characteristics of the P3HT thin films indicate typical p-channel operation with large current modulation, as expected (Figure 4 and Figure S2). The P3HT OFETs made without processing additives had a field-effect hole mobility of 0.0294 cm^2^ V^−1^ s^−1^ in the saturation regime. The on-state current increased with the use of the processing additives during formation of the P3HT thin films. When DIO was added to the P3HT solution, the maximum mobility of the OFETs increased to 0.114 cm^2^ V^−1^ s^−1^ (by a factor of 3.7). CN also produced a similar enhancement of the device performance, resulting in a mobility of 0.104 cm^2^ V^−1^ s^−1^. Other additives, like DPE and ODT, had relatively little impact on the field-effect mobility. The maximum mobility of the DPE- and ODT-treated P3HT films was 0.0382 cm^2^ V^−1^ s^−1^ and 0.0304 cm^2^ V^−1^ s^−1^, respectively. The average device parameters of the P3HT OFETs, with and without processing additives, are summarized in Table 1.

Notably, when CN and DIO were used as additives, the device performance improved drastically. We believe that this enhancement is related to changes in the molecular ordering of the P3HT thin films with the use of the processing additive and thermal annealing. A processing additive having a boiling point higher than that of the main solvent may provide sufficient time for ordering during the spin-coating step because of the lower rate of solvent evaporation, thereby increasing the crystallinity of the P3HT film and consequently improving the device performance [17]. In the case of ODT and DPE, the enhancement in the hole mobility of the P3HT thin films was relatively small (by a factor of 1.3 for ODT and 1.6 for DPE) compared to that induced by CN and DIO. To determine whether the additive also works as a post-treatment agent or not, we also compared the electrical characteristics of OFETs based on the P3HT thin films before and after dipping in DIO for 10 min (Figure S3). It was found that the DIO dipping procedure was ineffective for enhancing the performance of the P3HT OFETs. This observation suggests that the processing additive plays a role when dispersed in the polymer films, even with the use of a small amount.

**Figure 5:**
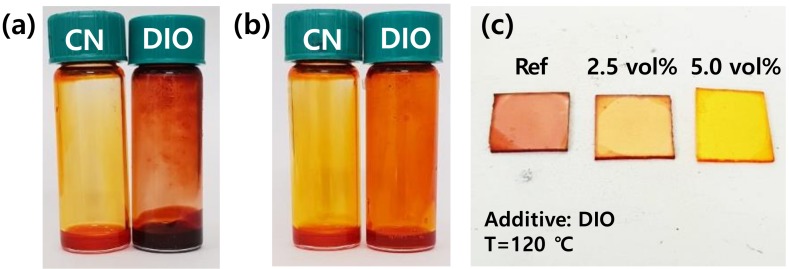
(**a**,**b**) P3HT solutions (10 mg mL^−1^) in CN and DIO as a solvent: (**a**) at 50 °C and (**b**) at 120 °C. (**c**) P3HT thin films at 120 °C, processed with various concentrations of DIO.

The effects of post-deposition thermal annealing of the P3HT thin films prepared with CN and DIO as processing additives were then investigated because these two chemicals are known to effectively enhance the field-effect mobility of polymer thin films after heat treatment. Heat treatment is a simple means of enhancing the field-effect mobility of polymer thin films [39,40,41]. Thermal energy can relax the polymer chains from a kinetically quenched state to a thermodynamically more stable and well-ordered state [39]. To compare CN and DIO, concentrated P3HT solutions were prepared in either CN or DIO without the main solvent, chloroform (Figure 5). It was observed that CN was a sufficiently good solvent for P3HT at 50 °C, whereas DIO was only able to dissolve the polymer at 120 °C. The P3HT thin films prepared with and without the DIO additive were then placed on a hot-plate at 120 °C, and the color of films changed from reddish-purple to orange, suggesting that P3HT dissolved somewhat at elevated temperature. The color became more yellowish for the P3HT films prepared from a stock solution with a higher DIO concentration. Based on this observation, it is proposed that the effects of processing additives may differ depending on the type of additive and the post-deposition heat treatment.

**Figure 6:**
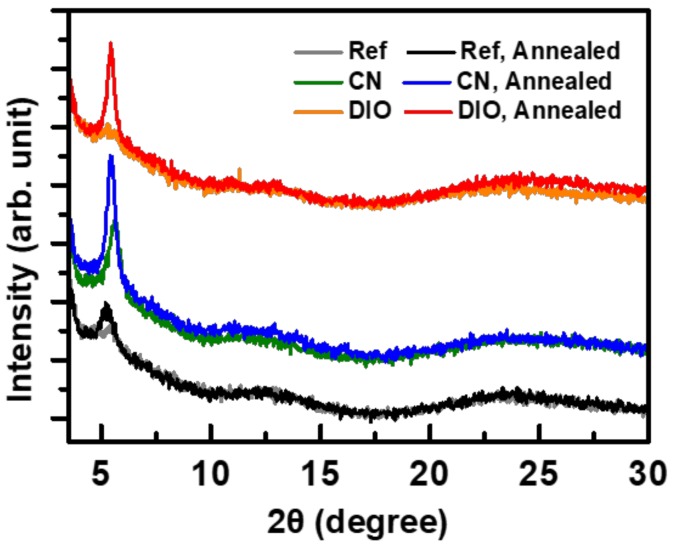
XRD profiles of P3HT thin films with/without additives and before/after thermal annealing at 120 °C for 30 min.

The XRD and AFM measurements demonstrate that the effect of thermal annealing on the morphology of P3HT thin films also differed depending on the type of additive. The XRD analyses provide insight into the effects of thermal annealing through the changes in the peak intensity and mean coherent length, *L*_C_ (Figure 6). In general, heat treatment enhanced the molecular ordering of the P3HT thin films, as evidenced by the changes in the *L*_C_. The mean coherent length of the P3HT films without any additive increased from 8.2 nm before annealing to 12.7 nm after annealing. With the use of DIO as an additive, the change in the *L*_C_ was more drastic (from 8.5 to 22.3 nm). On the other hand, the *L*_C_ of the films processed with CN was relatively large (13.1 nm) even before annealing. Thermal annealing of the CN-treated samples resulted in a small increase in the *L*_C_ to 17.8 nm. The increase in the *L*_C_ suggests a decrease in the stacking defects inside the polymer thin films.

It was also discovered that when P3HT was deposited from a solution in pure chloroform, the roughness decreased slightly from 1.2 nm before annealing to 0.8 nm after annealing (Figure S4). When DIO was added to the P3HT solution, *R*_q_ increased significantly, from 1.1 to 3.9 nm. At the same time, P3HT with CN as a processing additive had respective *R*_q_ values of 5.2 nm and 5.3 nm, before and after annealing. The difference between the thin films with and without heat treatment originates from the solubility of P3HT in the additive at relatively low temperature (see Figure 5). In the case of CN with a low vapor pressure and high solubility for P3HT at moderate temperature, the polymer thin film adopted relatively ordered molecular states during the spin-coating process before thermal annealing. The results of the XRD and AFM analyses show that post-deposition treatment has a great impact on molecular ordering of the P3HT thin films processed with additives, and that the degree of influence is more significant when the solubility of the polymer solubility varies significantly depending on the temperature (as for DIO). Thus, the present observations suggest that in the selection of processing additives for enhancing the performance of devices, the post-treatment conditions must be simultaneously considered.

**Figure 7:**
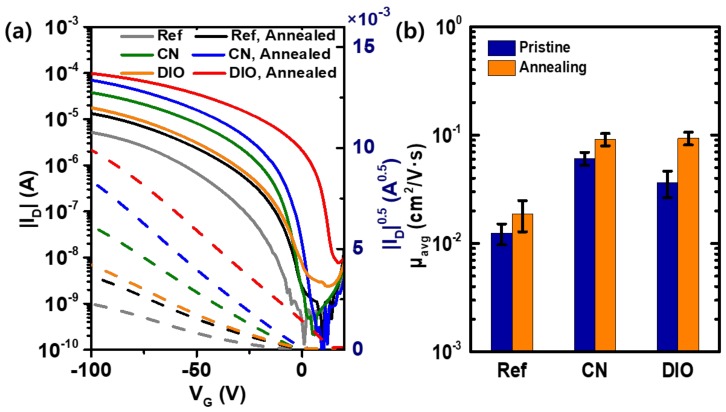
Effect of annealing and additive on OFET characteristics. (**a**) Transfer curves. (**b**) Comparison of hole mobility.

It is expected that the morphological evolution of the P3HT films after thermal processing in the presence of additives should affect the charge-transport properties of the films. Organic field-effect transistors were fabricated and characterized to evaluate the changes in the current–voltage curves, and, therefore, in the hole mobility of the P3HT films (Figure 7). Typical p-type characteristics were observed with a current modulation higher than 10^4^. For the as-spun P3HT films without additives, used as a control device in this work, the field-effect mobility was 0.0124 (±0.0027) cm^2^ V^−1^ s^−1^. This mobility increased by a factor of two to 0.0223 (±0.0051) cm^2^ V^−1^ s^−1^ after annealing at 120 °C for 30 min under nitrogen atmosphere. When the processing additives were used during coating of the thin films, the mobility increased sharply. The hole mobility of the OFETs based on the CN-treated P3HT films, before and after annealing, were 0.0609 (±0.0085) cm^2^ V^−1^ s^−1^ and 0.0914 (±0.0120) cm^2^ V^−1^ s^−1^, respectively. Annealing resulted in a 50% increase in the mobility when CN was used during film processing. The five-fold enhancement achieved with the CN additive, compared to that with the pristine films without additives, can be attributed to the morphological changes in the P3HT films both with and without thermal annealing. At moderate temperature, P3HT was sufficiently soluble in CN, as shown in Figure 5. This solubility provides molecular mobility of the P3HT chains, resulting in more crystalline local energetic minimum states.

The DIO-processed P3HT films showed a hole mobility of 0.0364 (±0.0099) cm^2^ V^−1^ s^−1^ without annealing. However, the value increased to 0.0937 (±0.0127) cm^2^ V^−1^ s^−1^ after annealing at 120 °C. The enhancement of the field-effect mobility upon heat treatment was more significant with DIO than with CN. This observation is consistent with the changes detected from AFM (Figure S4) and XRD (Figure 6). Since P3HT is only sufficiently soluble in DIO at high temperature (Figure 5), the P3HT films with a small amount of DIO could only undergo limited molecular rearrangement without high thermal energy. In this case, the films require thermal processing to adopt a morphology favorable to charge transport. Therefore, the processing conditions must be designed with consideration of the processing additives and post-deposition processing when fabricating OFETs and other organic electronic devices. Further studies on the combination of additives and post-deposition treatment on the thin films of newly developed polymer semiconductors with a high charge-carrier mobility, other than the benchmarking P3HT, would be valuable in development of better polymer semiconductors and processing methods.

## 4. Conclusions

It was demonstrated that the morphology and charge-carrier mobility of thin P3HT films are strongly related to the combination of processing additives and post-deposition thermal annealing. The solvent additive can induce relaxation of the polymer chains from kinetically trapped metastable states to the states with local minimum energy, as long as the polymer is sufficiently soluble at the processing temperature, as in the case of CN. If the polymer is only soluble in the additive at a higher temperature, as in the case of DIO, heat treatment after film deposition is essential to induce better molecular ordering for achieving enhanced charge transport in polymer thin films. The morphological and electrical investigation in this work suggest that careful combination of the processing solvent systems and post-deposition processing are critical for achieving high charge-carrier mobility, and therefore high performance, in thin film devices. Design of the processing conditions for polymer films and organic electronic devices must be performed with consideration of various aspects of the processing variables, because various additives may act in different ways depending on the interaction with constituent organic semiconductors.

## Figures and Tables

**Table 1:** Properties of processing additives, morphological parameters of P3HT thin films, and electrical parameters of thermally annealed P3HT OFETs.

Additive	M.W. ^a^ (g/mol)	B.P. ^b^ (°C)	*P*_vapor_^c^ (mmHg)	*R*_q_^d^ (nm)	*d*_(100)_^e^ (nm)	*L*_C_^f^ (nm)	*μ*_max_^g^ (cm^2^/Vs)	*μ*_avg_^h^ (cm^2^/Vs)	*V*_th_^i^ (V)
-	-	-	-	0.8	1.67	13.2	0.0294	0.0223 (±0.0051)	−20.6 (±3.9)
1,8-Octanedithiol(ODT)	178.4	270	0.012	3.8	1.64	16.7	0.0304	0.0292 (±0.0010)	−12.7 (±7.9)
Diphenyl ether(DPE)	170.2	259	0.02	5.0	1.64	16.8	0.0382	0.0359 (±0.0032)	−18.0 (±6.4)
1-Chloronaphthalene(CN)	162.6	263	0.029	5.3	1.63	18.2	0.104	0.0914 (±0.0120)	2.2 (±11.6)
1,8-Diiodooctane(DIO)	366.0	332	0.0003	3.9	1.63	24.8	0.114	0.0937 (±0.0127)	7.58 (±9.1)

^a^ Molecular weight; ^b^ Boiling point; ^c^ Vapor pressure at 25 °C; ^d^ Root mean square roughness calculated from AFM images (5 × 5 µm^2^); ^e^ d_(100)_-spacing; ^f^ Mean coherent length; ^g^ Maximum field-effect hole mobility; ^h^ Average field-effect hole mobility with a standard deviation; ^i^ Average threshold voltage with standard deviation.

## Supplementary Materials

The following are available online at http://www.mdpi.com/2073-4360/11/1/112/s1: Figures S1–S5. XRD, output curves, dipping experiment results, and AFM images.
