# Supplementary material for: Synergistic Effects of Processing Additives and Thermal Annealing on Nanomorphology and Hole Mobility of Poly(3-hexylthiophene) Thin Films
Source: Polymers (Basel). 2019 Jan 10;11(1):112. doi: 10.3390/polym11010112 (PMC6401865; doi:10.3390/polym11010112)
Supplement: Supplementary file 1 [file polymers-11-00112-s001.pdf]

Supporting Information

Synergistic Effects of Processing Additives and Thermal Annealing on Nanomorphology and Hole Mobility of Poly(3-hexylthiophene) Thin Films

Min Soo Park and Felix Sunjoo Kim*

School of Chemical Engineering and Materials Science, Chung-Ang University, Seoul, 06974, Republic of Korea

**Corresponding author email: fskim@cau.ac.kr*

Figure S1. XRD profiles of P3HT thin films fabricated with various additives. Films were thermally annealed at 120 °C for 30 min.

Figure S2. Output curves of P3HT field-effect transistors processed without additives (a) and with ODT (b), DPE (c), CN (d), and DIO (e). Films were thermally annealed at 120 °C for 30 min.

Figure S3. Characteristics of OFET fabricated by dipping method: (a) transfer curves for OFETs. (b) Comparison of hole mobility. (c) Output curves of P3HT without dipping procedure. (d) Output curves of P3HT after dipping in DIO. Films were thermally annealed at 120 °C for 30 min.

Figure S4. AFM topography images: (a,b) P3HT films cast from a solution in chloroform. (c,d) P3HT films cast from a chloroform solution containing 2.5 vol% of CN. (e,f) P3HT films cast from a chloroform solution containing 2.5 vol% of DIO; (a,c,e) thin films before thermal annealing. (b,d,f) Thin-films after thermal annealing at 120 °C for 30 min.

Figure S5. Output curves of P3HT field-effect transistors: (a,b) P3HT films cast from pure solvent. (c,d) P3HT films cast from solvent containing 2.5 vol% CN. (e,f) P3HT films cast from solvent containing 2.5 vol% DIO. Films in (a,c,e) were not annealed. Films in (b,d,f) were annealed at 120 °C for 30 min.

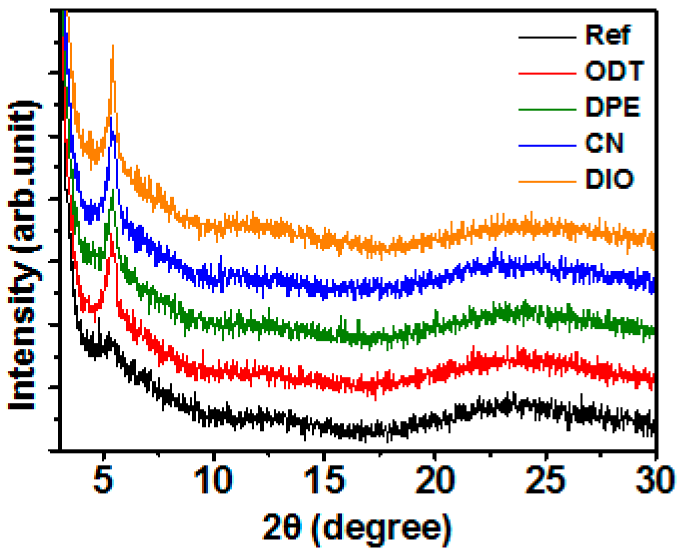

Figure S1. XRD profiles of P3HT thin films fabricated with various additives. Films were thermally annealed at 120 °C for 30 min.

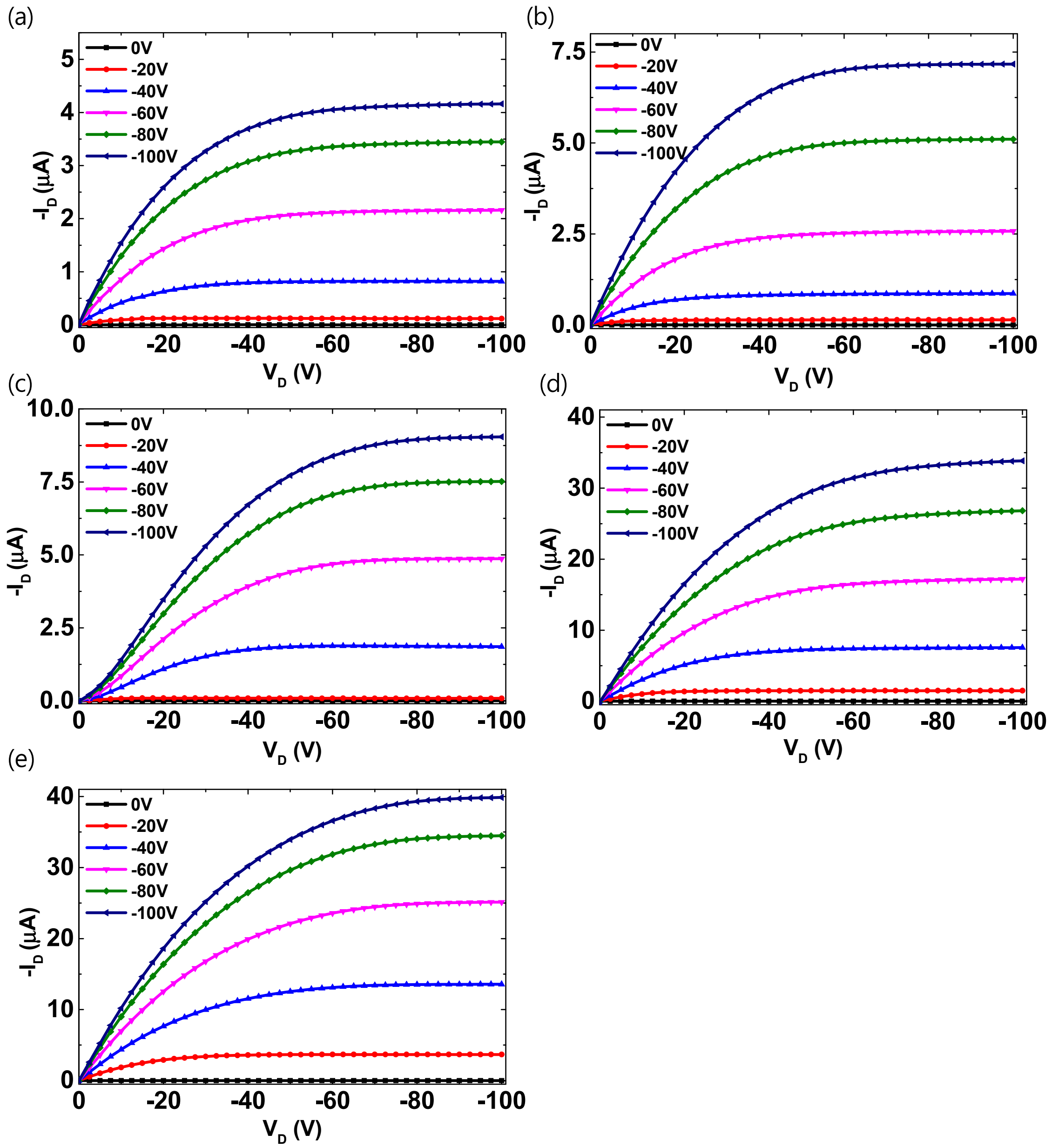

Figure S2. Output curves of P3HT field-effect transistors processed without additives (a) and with ODT (b), DPE (c), CN (d), and DIO (e). Films were thermally annealed at 120 °C for 30 min.

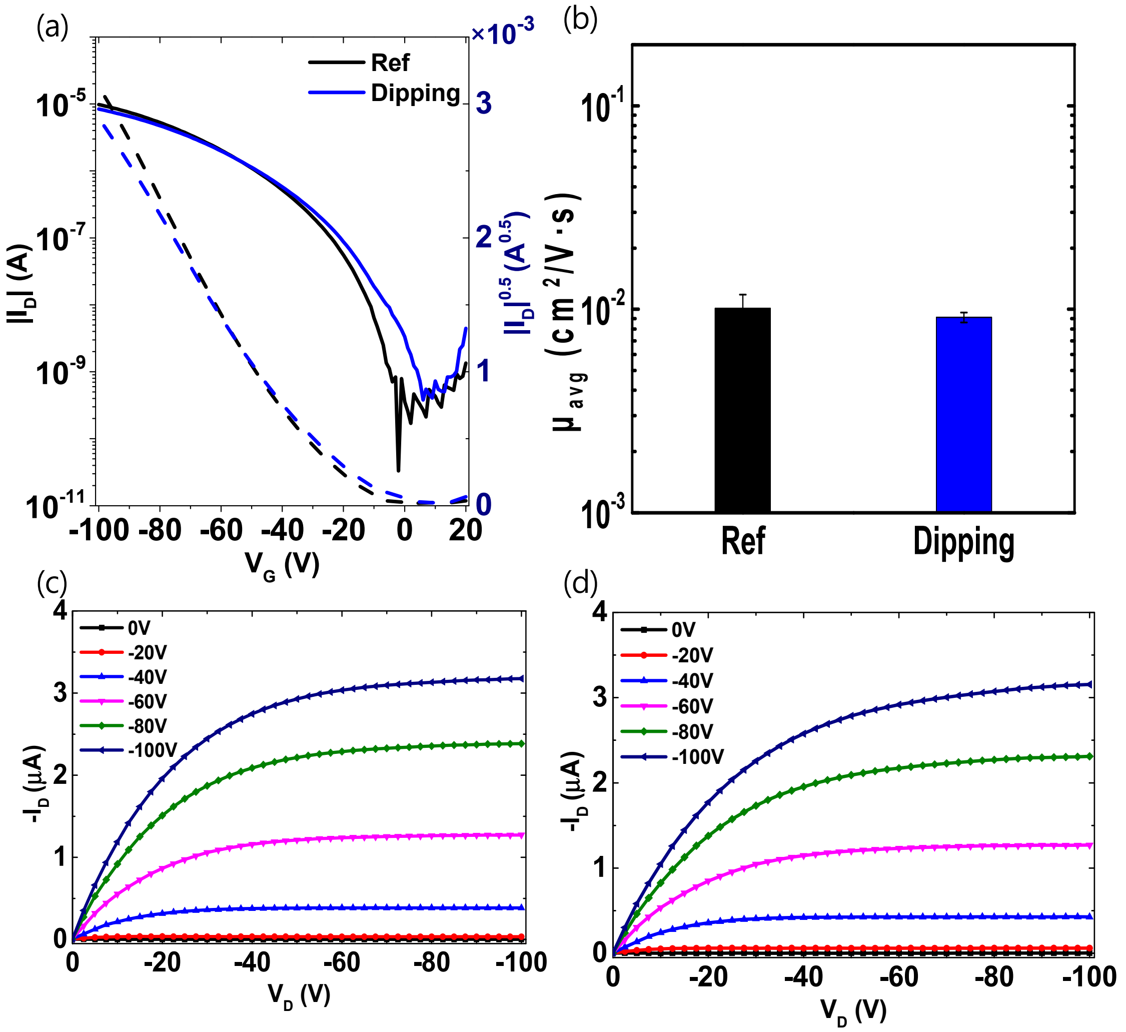

Figure S3. Characteristics of OFET fabricated by dipping method: (a) transfer curves of OFETs. (b) Comparison of hole mobility. (c) Output curves of P3HT without dipping procedure. (d) Output curves of P3HT after dipping in DIO. Films were thermally annealed at 120 °C for 30 min.

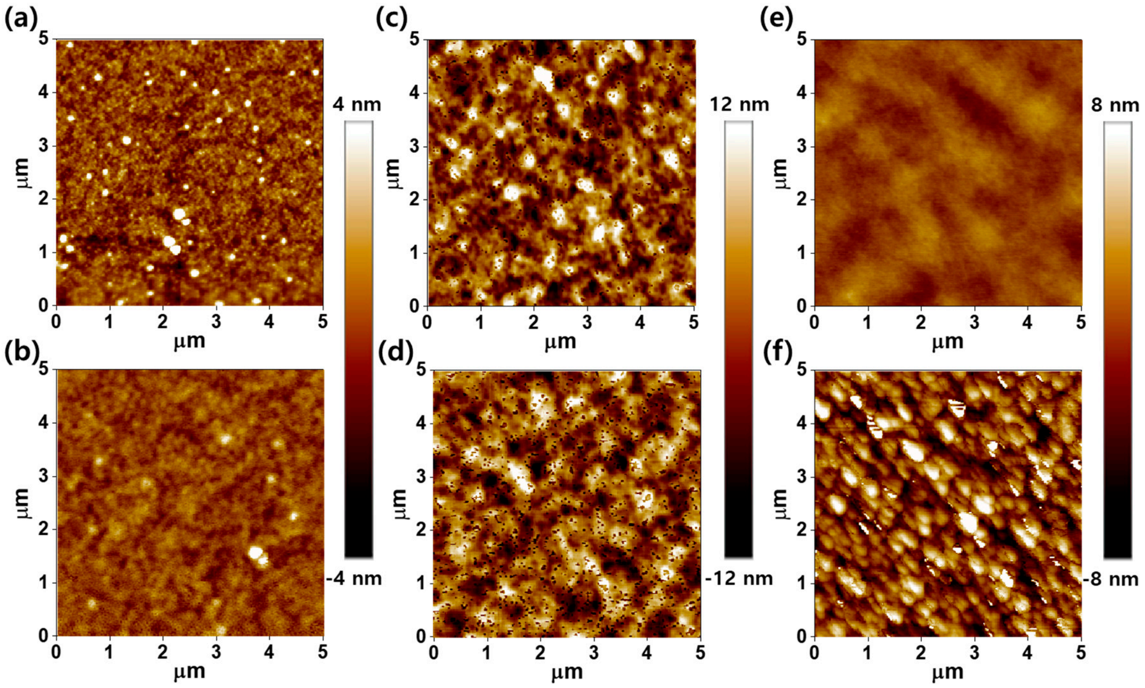

Figure S4. AFM topography images: (a,b) P3HT films cast from a solution in chloroform. (c,d) P3HT films cast from chloroform solution containing 2.5 vol% of CN. (e,f) P3HT films cast from chloroform solution containing 2.5 vol% of DIO; (a,c,e) thin films before thermal annealing. (b,d,f) Thin films after thermal annealing at 120 °C for 30 min.

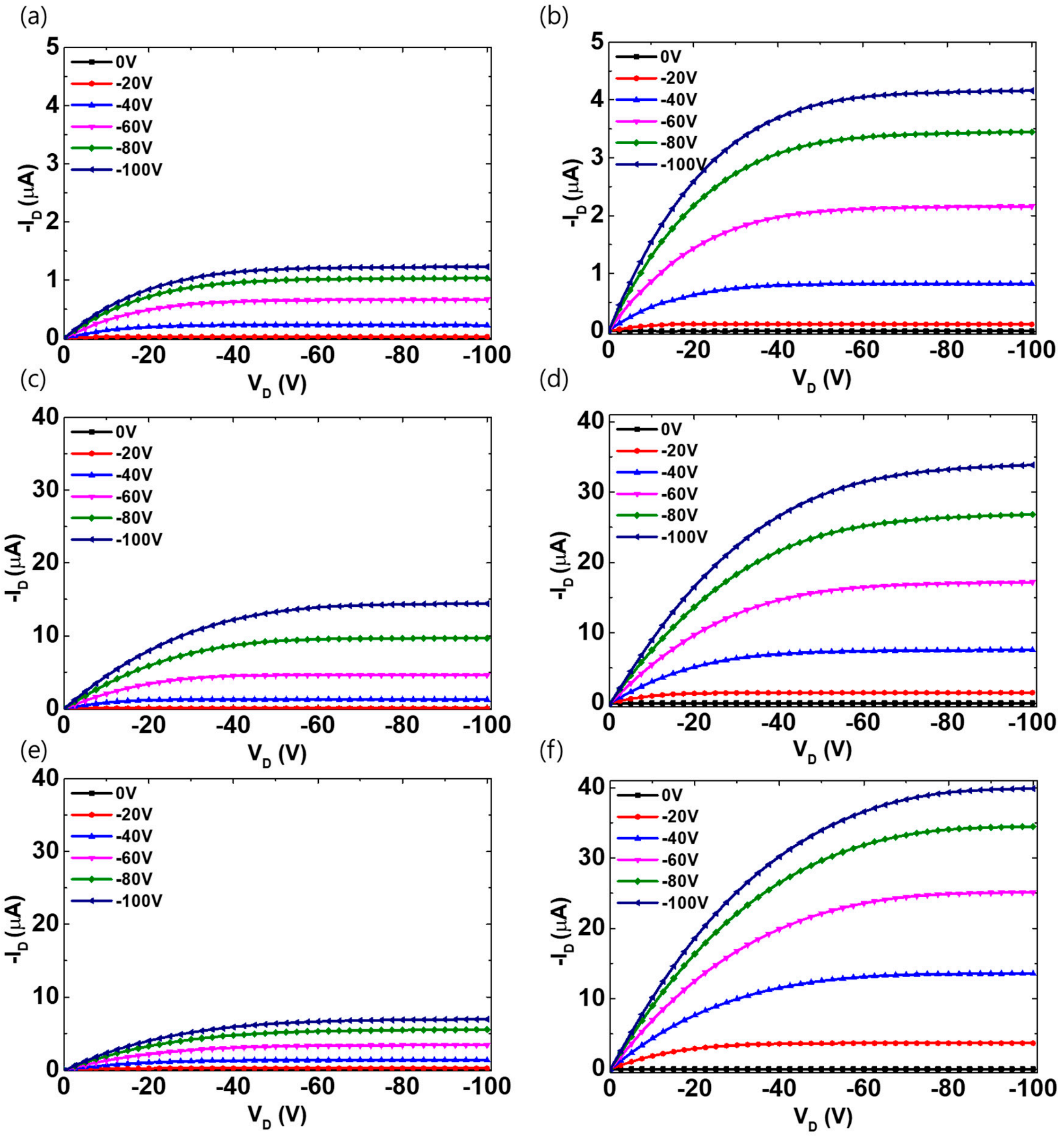

Figure S5. Output curves of P3HT field-effect transistors: (a,b) P3HT films cast from pure solvent. (c,d) P3HT films cast from solvent containing 2.5 vol% CN. (e,f) P3HT films cast from solvent containing 2.5 vol% DIO. Films in (a,c,e) were not annealed. Films in (b,d,f) were annealed at 120 °C for 30 min.
